# (μ-6-Oxido-4-oxo-1,2-dihydro­pyrimidine-5-carboxyl­ato-κ^4^ *O* ^5^,*O* ^6^:*O* ^2^,*N* ^3^)bis­[aquabis­(4-oxido-2-oxo-1,2-dihydro­pyrimidin-3-ium-5-carboxyl­ato-κ^2^ *O* ^4^,*O* ^5^)­(1,10-phenanthroline-κ^2^ *N*,*N*′)neodymium(III)] hexa­hydrate

**DOI:** 10.1107/S1600536808001499

**Published:** 2008-01-25

**Authors:** Hui-Hui Xing, Zi-Lu Chen, Seik Weng Ng

**Affiliations:** a College of Chemistry and Chemical Engineering, Guangxi Normal University, Gulin 541004, People’s Republic of China; b Department of Chemistry, University of Malaya, 50603, Kuala Lumpur, Malaysia

## Abstract

The water-coordinated neodymium(III) atom in the centrosymmetric title compound, [Nd_2_(C_5_H_2_N_2_O_4_)(C_5_H_3_N_2_O_4_)_4_(C_12_H_8_N_2_)_2_(H_2_O)_2_]·6H_2_O is chelated by a 1,10-phenanthroline heterocycle and two 2,4-dihydroxy­pyrimidine-5-carboxyl­ate dianions. Two tris-chelated water-coordinated units are bridged by a 2,4-dihydroxy­pyrimidine-5-carboxyl­ate dianion, which is disordered about a center of inversion. The metal center has a monocapped square-anti­prismatic coordination.

## Related literature

The title neodymium compound is isostructural with the praseodymium analog (Sun & Jin, 2004 ▶). For a related structure, see: Xing *et al.* (2008 ▶).
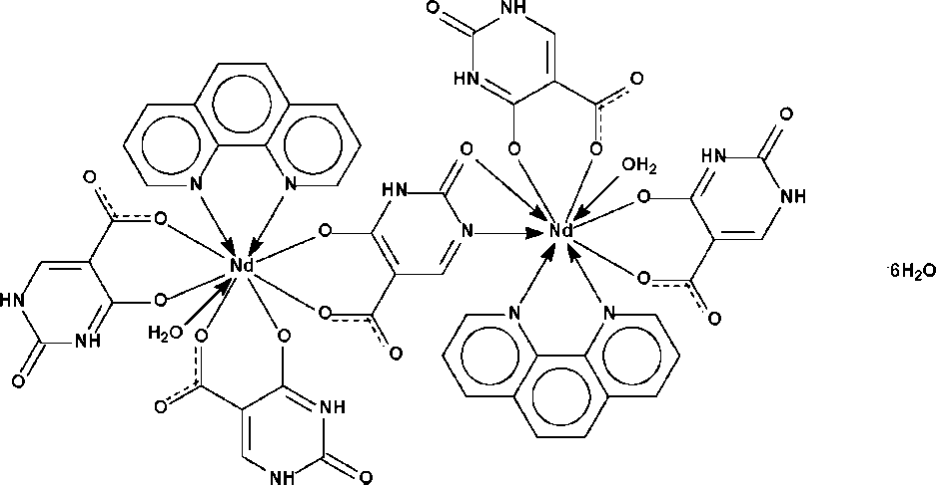


## Experimental

### 

#### Crystal data

[Nd_2_(C_5_H_2_N_2_O_4_)(C_5_H_3_N_2_O_4_)_4_(C_12_H_8_N_2_)_2_(H_2_O)_2_]·6H_2_O
*M* *_r_* = 1567.48
Triclinic, 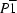

*a* = 10.1980 (7) Å
*b* = 12.8896 (9) Å
*c* = 13.3383 (9) Å
α = 118.711 (1)°
β = 90.010 (1)°
γ = 108.612 (1)°
*V* = 1432.25 (17) Å^3^
*Z* = 1
Mo *K*α radiation
μ = 1.90 mm^−1^
*T* = 295 (2) K
0.10 × 0.04 × 0.02 mm

#### Data collection

Bruker APEXII diffractometer
Absorption correction: multi-scan (*SADABS*; Sheldrick, 1996 ▶) *T*_min_ = 0.724, *T*_max_ = 0.963
12405 measured reflections
6377 independent reflections
5349 reflections with *I* > 2σ(*I*)
*R*_int_ = 0.039

#### Refinement

*R*[*F* ^2^ > 2σ(*F* ^2^)] = 0.054
*wR*(*F* ^2^) = 0.136
*S* = 1.04
6377 reflections
454 parameters
84 restraints
H-atom parameters constrained
Δρ_max_ = 1.23 e Å^−3^
Δρ_min_ = −0.90 e Å^−3^

### 

Data collection: *APEX2* (Bruker, 2006 ▶); cell refinement: *SAINT* (Bruker, 2006 ▶); data reduction: *SAINT*; method used to solve structure: atomic coordinates taken from the Pr analog (Sun & Jin, 2004 ▶); program(s) used to refine structure: *SHELXL97* (Sheldrick, 2008 ▶); molecular graphics: *X-SEED* (Barbour, 2001 ▶); software used to prepare material for publication: *publCIF* (Westrip, 2008 ▶).

## Supplementary Material

Crystal structure: contains datablocks global, I. DOI: 10.1107/S1600536808001499/ci2552sup1.cif

Structure factors: contains datablocks I. DOI: 10.1107/S1600536808001499/ci2552Isup2.hkl

Additional supplementary materials:  crystallographic information; 3D view; checkCIF report

## Figures and Tables

**Table:** 

Nd1—O2	2.526 (4)
Nd1—O3	2.502 (9)
Nd1—O5	2.392 (4)
Nd1—O6	2.575 (4)
Nd1—O9	2.341 (4)
Nd1—O10	2.526 (4)
Nd1—O1*W*	2.475 (4)
Nd1—N7	2.638 (5)
Nd1—N8	2.725 (5)

**Table:** 

O2—Nd1—O3	67.5 (2)
O2—Nd1—O5	71.6 (1)
O2—Nd1—O6	123.5 (1)
O2—Nd1—O9	127.7 (1)
O2—Nd1—O10	71.8 (1)
O2—Nd1—O1*W*	136.6 (2)
O2—Nd1—N7	70.6 (2)
O2—Nd1—N8	111.7 (2)
O3—Nd1—O5	138.7 (2)
O3—Nd1—O6	133.6 (3)
O3—Nd1—O9	68.6 (3)
O3—Nd1—O10	76.5 (3)
O3—Nd1—O1*W*	138.2 (3)
O3—Nd1—N7	87.9 (3)
O3—Nd1—N8	65.6 (3)
O5—Nd1—O6	67.9 (1)
O5—Nd1—O9	139.7 (2)
O5—Nd1—O10	85.9 (1)
O5—Nd1—O1*W*	78.2 (2)
O5—Nd1—N7	83.8 (2)
O5—Nd1—N8	138.8 (2)
O6—Nd1—O9	72.6 (1)
O6—Nd1—O10	67.7 (1)
O6—Nd1—O1*W*	69.0 (1)
O6—Nd1—N7	138.2 (2)
O6—Nd1—N8	124.8 (2)
O9—Nd1—O10	71.5 (1)
O9—Nd1—O1*W*	95.5 (2)
O9—Nd1—N7	133.8 (2)
O9—Nd1—N8	72.8 (2)
O10—Nd1—O1*W*	136.7 (2)
O10—Nd1—N7	142.5 (2)
O10—Nd1—N8	135.1 (2)
O1*W*—Nd1—N7	75.78 (16)
O1*W*—Nd1—N8	72.93 (16)
N7—Nd1—N8	61.14 (16)

**Fig. 1.**
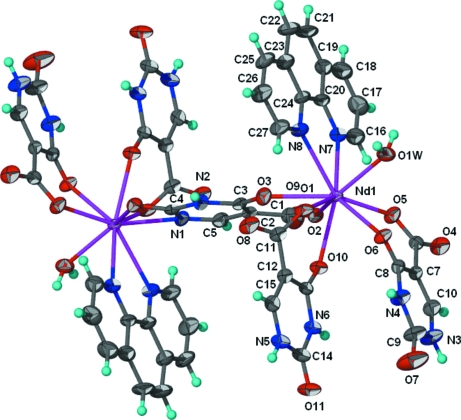
Displacement ellipsoid plot of Nd2(C12H8N2)2(C5H2N2O4)(C5H3N2O4)4(H2O)2.6H2O drawn at the 50% probability level. Unlabelled atoms are related to labelled atoms by the symmetry operation (1 - x, 1 - y, 1 - z). Lattice water molecules have been omitted for clarity. Only one disorder component is shown.

**Fig. 2.**
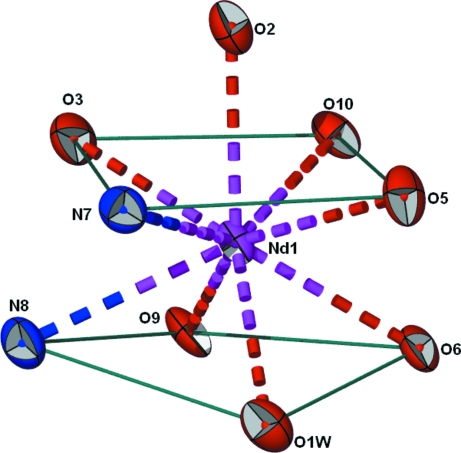
Geometry of Nd.

## supplementary crystallographic information

### Comment

The 1,10-phenanthroline-chelated rare-earth compound, Pr_2_(C_12_H_8_N_2_)_2_(C_5_H_2_N_2_O_4_)(C_5_H_3_N_2_O_4_)_4_(H_2_O)_2_– ^.^3H_2_O, represents the first example of dinculear lanthanum derivatives of 2,4-dihydroxpyrimidine-5-carboxylic acid (Sun & Jin, 2004). The present neodymium compound is isostructural with the praseodymium analog.

### Experimental

2,4-Dihydroxypyrimidine-5-carboxylic acid (0.044 g, 0.25 mmol), neodymium trichloride hexahydrate (0.090 g, 0.25 mmol), 1,10-phenanthroline (0.050 g, 0.25 mmol), sodium hydroxide (0.010 g, 0.25 mmol) and water (15 ml) was sealed in a 25-ml, Teflon-lined, stainless-steel Parr bomb. The bomb was heated to 383 K for 120 h. It was then cooled over 48 h to give lilac crystals in 70% yield. CH&N elemental analysis. Found (Calculated) for C_49_H_46_N_14_O_28_Nd_2_: C 37.37, H 3.07, N 12.62% (37.54, 2.96, 12.51%).

### Refinement

The bridging dianion is disordered about a center of inversion; all atoms were assigned only half site-occupancy except for O2, which had full site occupancy. The pyrimidine ring was refined as a rigid hexagon of 1.39 Å sides.

Carbon-bound H atoms were generated geometrically, and were included in the refinements in the riding model approximation, as well the nitrogen-bound ones. For the water molecules, only those on the coordinated water were were placed in chemically sensible positions on the basis of hydrogen bonding interactions. TWo of the lattice water molecules are each disordered over two positions. The displacement parameters of the two components were restrained to be equal. The H atoms bound to the lattice water molecules could not be located. The final difference Fourier map had a large peak near Nd1.

### Crystal data

**Table:**

[Nd_2_(C_5_H_2_N_2_O_4_)(C_5_H_3_N_2_O_4_)_4_(C_12_H_8_N_2_)_2_(H_2_O)_2_]·6H_2_O	*Z* = 1
*M**_r_* = 1567.48	*F*_000_ = 782
Triclinic, *P*1	*D*_x_ = 1.817 Mg m^−^^3^
Hall symbol: -P 1	Mo *K*α radiation λ = 0.71073 Å
*a* = 10.1980 (7) Å	Cell parameters from 2901 reflections
*b* = 12.8896 (9) Å	θ = 2.3–22.7º
*c* = 13.3383 (9) Å	µ = 1.90 mm^−^^1^
α = 118.711 (1)º	*T* = 295 (2) K
β = 90.010 (1)º	Prism, lilac
γ = 108.612 (1)º	0.10 × 0.04 × 0.02 mm
*V* = 1432.25 (17) Å^3^	

### Data collection

**Table:**

Bruker APEXII diffractometer	6377 independent reflections
Radiation source: fine-focus sealed tube	5349 reflections with *I* > 2σ(*I*)
Monochromator: graphite	*R*_int_ = 0.039
*T* = 295(2) K	θ_max_ = 27.5º
φ and ω scans	θ_min_ = 1.8º
Absorption correction: multi-scan(SADABS; Sheldrick, 1996)	*h* = −13→13
*T*_min_ = 0.724, *T*_max_ = 0.963	*k* = −16→16
12405 measured reflections	*l* = −17→17

### Refinement

**Table:**

Refinement on *F*^2^	Secondary atom site location: difference Fourier map
Least-squares matrix: full	Hydrogen site location: inferred from neighbouring sites
*R*[*F*^2^ > 2σ(*F*^2^)] = 0.054	H-atom parameters constrained
*wR*(*F*^2^) = 0.136	*w* = 1/[σ^2^(*F*_o_^2^) + (0.0735*P*)^2^ + 0.4633*P*] where *P* = (*F*_o_^2^ + 2*F*_c_^2^)/3
*S* = 1.04	(Δ/σ)_max_ = 0.001
6377 reflections	Δρ_max_ = 1.23 e Å^−^^3^
454 parameters	Δρ_min_ = −0.90 e Å^−^^3^
84 restraints	Extinction correction: none
Primary atom site location: structure-invariant direct methods	

### Fractional atomic coordinates and isotropic or equivalent isotropic displacement parameters (Å^2^)

**Table:**

	*x*	*y*	*z*	*U*_iso_*/*U*_eq_	Occ. (<1)
Nd1	0.26520 (3)	0.27712 (3)	0.14686 (3)	0.02555 (12)	
O4	0.2306 (5)	−0.1369 (4)	−0.0511 (5)	0.0486 (12)	
O5	0.2203 (5)	0.0542 (4)	0.0456 (4)	0.0400 (11)	
O6	0.3131 (5)	0.1959 (4)	−0.0621 (4)	0.0349 (10)	
O7	0.6565 (7)	0.1207 (7)	−0.2689 (7)	0.090 (2)	
O8	0.5206 (5)	0.6553 (4)	0.2061 (5)	0.0490 (13)	
O9	0.3726 (4)	0.4550 (4)	0.1277 (4)	0.0370 (10)	
O10	0.5209 (4)	0.3057 (4)	0.1378 (4)	0.0334 (9)	
O11	0.9276 (5)	0.3690 (5)	0.0047 (5)	0.0535 (14)	
O1W	0.0466 (5)	0.1916 (4)	0.0068 (4)	0.0442 (11)	
H1W1	−0.0305	0.1557	0.0212	0.066*	
H1W2	0.0389	0.2526	0.0013	0.066*	
O2W	−0.0203 (12)	0.1690 (10)	−0.2139 (10)	0.156 (4)	
O3W	0.212 (2)	0.192 (2)	−0.3809 (18)	0.149 (5)	0.50
O4W	0.483 (2)	0.789 (2)	0.4468 (18)	0.155 (5)	0.50
O3'	0.144 (2)	−0.0260 (19)	−0.5588 (18)	0.149 (5)	0.50
O4'	0.418 (2)	0.918 (2)	0.4944 (18)	0.155 (5)	0.50
N3	0.5421 (7)	−0.0189 (6)	−0.2106 (6)	0.0512 (16)	
H3N	0.5912	−0.0654	−0.2433	0.061*	
N4	0.4768 (6)	0.1499 (5)	−0.1699 (5)	0.0415 (13)	
H4N	0.4830	0.2124	−0.1797	0.050*	
N5	0.8306 (5)	0.5190 (5)	0.0582 (5)	0.0373 (13)	
H5N	0.8986	0.5647	0.0416	0.045*	
N6	0.7237 (5)	0.3425 (5)	0.0743 (5)	0.0325 (11)	
H6N	0.7251	0.2736	0.0689	0.039*	
N7	0.0616 (5)	0.2175 (5)	0.2535 (5)	0.0359 (12)	
N8	0.1220 (6)	0.4365 (5)	0.2479 (5)	0.0404 (13)	
C6	0.2655 (6)	−0.0264 (6)	−0.0300 (5)	0.0302 (13)	
C7	0.3654 (6)	0.0150 (5)	−0.0962 (5)	0.0282 (12)	
C8	0.3800 (6)	0.1251 (5)	−0.1048 (5)	0.0291 (12)	
C9	0.5642 (8)	0.0857 (8)	−0.2206 (7)	0.053 (2)	
C10	0.4462 (7)	−0.0521 (6)	−0.1512 (6)	0.0386 (15)	
H10	0.4356	−0.1249	−0.1482	0.046*	
C11	0.4953 (7)	0.5402 (6)	0.1568 (5)	0.0319 (13)	
C12	0.6137 (6)	0.4941 (5)	0.1250 (5)	0.0300 (13)	
C13	0.6123 (6)	0.3775 (5)	0.1135 (5)	0.0279 (12)	
C14	0.8335 (6)	0.4081 (6)	0.0429 (6)	0.0379 (15)	
C15	0.7258 (6)	0.5607 (6)	0.0983 (5)	0.0338 (14)	
H15	0.7303	0.6383	0.1081	0.041*	
C16	0.0321 (8)	0.1146 (7)	0.2595 (6)	0.0479 (17)	
H16	0.0852	0.0640	0.2260	0.057*	
C17	−0.0746 (8)	0.0765 (8)	0.3131 (7)	0.058 (2)	
H17	−0.0915	0.0024	0.3154	0.069*	
C18	−0.1540 (8)	0.1494 (9)	0.3626 (7)	0.061 (2)	
H18	−0.2255	0.1251	0.3989	0.073*	
C19	−0.1288 (7)	0.2575 (8)	0.3586 (6)	0.0489 (18)	
C20	−0.0184 (6)	0.2911 (7)	0.3026 (5)	0.0382 (15)	
C21	−0.2097 (8)	0.3371 (10)	0.4055 (7)	0.067 (3)	
H21	−0.2834	0.3149	0.4410	0.080*	
C22	−0.1824 (8)	0.4419 (9)	0.3998 (7)	0.062 (2)	
H22	−0.2382	0.4906	0.4299	0.074*	
C23	−0.0688 (8)	0.4805 (8)	0.3481 (6)	0.0499 (18)	
C24	0.0120 (7)	0.4047 (6)	0.2980 (5)	0.0376 (15)	
C25	−0.0328 (9)	0.5927 (8)	0.3471 (7)	0.059 (2)	
H25	−0.0849	0.6447	0.3789	0.071*	
C26	0.0792 (9)	0.6270 (8)	0.2994 (7)	0.059 (2)	
H26	0.1058	0.7036	0.3005	0.071*	
C27	0.1537 (8)	0.5466 (7)	0.2491 (7)	0.0483 (18)	
H27	0.2284	0.5700	0.2149	0.058*	
O1	0.3919 (11)	0.1911 (9)	0.4221 (9)	0.059 (3)	0.50
O2	0.3542 (4)	0.2234 (4)	0.2860 (4)	0.0383 (10)	
O3	0.3932 (12)	0.4700 (9)	0.3390 (8)	0.042 (3)	0.50
C1	0.3988 (6)	0.2642 (7)	0.3854 (7)	0.034 (3)	0.50
C2	0.4626 (9)	0.4027 (5)	0.4640 (6)	0.021 (3)	0.50
C3	0.4558 (9)	0.4923 (6)	0.4366 (5)	0.032 (3)	0.50
N2	0.5211 (9)	0.6211 (6)	0.5170 (6)	0.028 (3)	0.50
H2N	0.5169	0.6766	0.5001	0.034*	0.50
C4	0.5931 (8)	0.6601 (5)	0.6250 (6)	0.033 (3)	0.50
N1	0.5999 (9)	0.5705 (8)	0.6524 (5)	0.033 (3)	0.50
C5	0.5346 (9)	0.4417 (7)	0.5719 (7)	0.040 (3)	0.50
H5	0.5391	0.3817	0.5903	0.048*	0.50

### Atomic displacement parameters (Å^2^)

**Table:**

	*U*^11^	*U*^22^	*U*^33^	*U*^12^	*U*^13^	*U*^23^
Nd1	0.02497 (18)	0.02326 (18)	0.03442 (19)	0.01369 (13)	0.01123 (12)	0.01601 (14)
O4	0.057 (3)	0.030 (2)	0.071 (3)	0.022 (2)	0.022 (3)	0.030 (3)
O5	0.051 (3)	0.025 (2)	0.047 (3)	0.015 (2)	0.021 (2)	0.019 (2)
O6	0.048 (3)	0.033 (2)	0.041 (2)	0.027 (2)	0.019 (2)	0.023 (2)
O7	0.103 (5)	0.095 (5)	0.133 (6)	0.066 (4)	0.085 (5)	0.084 (5)
O8	0.059 (3)	0.028 (2)	0.074 (3)	0.024 (2)	0.031 (3)	0.031 (2)
O9	0.038 (2)	0.035 (2)	0.057 (3)	0.026 (2)	0.021 (2)	0.030 (2)
O10	0.030 (2)	0.032 (2)	0.052 (3)	0.0154 (18)	0.0149 (19)	0.029 (2)
O11	0.038 (3)	0.046 (3)	0.100 (4)	0.025 (2)	0.036 (3)	0.048 (3)
O1W	0.035 (2)	0.046 (3)	0.058 (3)	0.021 (2)	0.011 (2)	0.026 (2)
O2W	0.174 (8)	0.147 (7)	0.156 (7)	0.074 (6)	0.026 (6)	0.075 (6)
O3W	0.195 (10)	0.126 (8)	0.143 (8)	0.064 (7)	0.005 (7)	0.077 (7)
O4W	0.177 (10)	0.163 (9)	0.127 (8)	0.035 (7)	0.036 (7)	0.092 (7)
O3'	0.195 (10)	0.126 (8)	0.143 (8)	0.064 (7)	0.005 (7)	0.077 (7)
O4'	0.177 (10)	0.163 (9)	0.127 (8)	0.035 (7)	0.036 (7)	0.092 (7)
N3	0.063 (4)	0.052 (4)	0.070 (4)	0.042 (3)	0.036 (3)	0.041 (3)
N4	0.051 (3)	0.037 (3)	0.057 (4)	0.025 (3)	0.027 (3)	0.034 (3)
N5	0.029 (3)	0.035 (3)	0.064 (4)	0.012 (2)	0.016 (3)	0.036 (3)
N6	0.028 (3)	0.027 (3)	0.053 (3)	0.011 (2)	0.012 (2)	0.027 (3)
N7	0.033 (3)	0.040 (3)	0.040 (3)	0.016 (2)	0.010 (2)	0.023 (3)
N8	0.038 (3)	0.044 (3)	0.045 (3)	0.025 (3)	0.015 (3)	0.021 (3)
C6	0.029 (3)	0.028 (3)	0.037 (3)	0.012 (2)	0.005 (2)	0.018 (3)
C7	0.030 (3)	0.024 (3)	0.031 (3)	0.013 (2)	0.003 (2)	0.013 (3)
C8	0.031 (3)	0.023 (3)	0.032 (3)	0.011 (2)	0.007 (2)	0.013 (3)
C9	0.057 (5)	0.060 (5)	0.067 (5)	0.037 (4)	0.037 (4)	0.040 (4)
C10	0.046 (4)	0.035 (3)	0.046 (4)	0.021 (3)	0.012 (3)	0.024 (3)
C11	0.041 (4)	0.033 (3)	0.038 (3)	0.020 (3)	0.016 (3)	0.026 (3)
C12	0.033 (3)	0.027 (3)	0.036 (3)	0.012 (3)	0.009 (3)	0.019 (3)
C13	0.023 (3)	0.027 (3)	0.035 (3)	0.009 (2)	0.005 (2)	0.017 (3)
C14	0.027 (3)	0.034 (3)	0.061 (4)	0.012 (3)	0.011 (3)	0.030 (3)
C15	0.037 (3)	0.023 (3)	0.045 (4)	0.010 (3)	0.006 (3)	0.020 (3)
C16	0.043 (4)	0.049 (4)	0.053 (4)	0.017 (3)	0.014 (3)	0.027 (4)
C17	0.050 (5)	0.060 (5)	0.063 (5)	0.010 (4)	0.014 (4)	0.038 (4)
C18	0.043 (4)	0.077 (6)	0.057 (5)	0.013 (4)	0.018 (4)	0.036 (5)
C19	0.036 (4)	0.064 (5)	0.035 (4)	0.014 (4)	0.011 (3)	0.019 (4)
C20	0.028 (3)	0.054 (4)	0.030 (3)	0.020 (3)	0.010 (3)	0.016 (3)
C21	0.041 (4)	0.104 (8)	0.054 (5)	0.037 (5)	0.031 (4)	0.032 (5)
C22	0.048 (5)	0.086 (7)	0.061 (5)	0.044 (5)	0.026 (4)	0.031 (5)
C23	0.047 (4)	0.058 (5)	0.041 (4)	0.031 (4)	0.009 (3)	0.015 (4)
C24	0.035 (3)	0.047 (4)	0.032 (3)	0.027 (3)	0.010 (3)	0.014 (3)
C25	0.063 (5)	0.063 (5)	0.051 (5)	0.047 (4)	0.016 (4)	0.014 (4)
C26	0.065 (5)	0.050 (5)	0.070 (5)	0.039 (4)	0.014 (4)	0.025 (4)
C27	0.053 (4)	0.040 (4)	0.064 (5)	0.030 (4)	0.022 (4)	0.027 (4)
O1	0.076 (6)	0.042 (5)	0.057 (6)	0.020 (5)	0.013 (5)	0.025 (4)
O2	0.036 (2)	0.028 (2)	0.048 (3)	0.0158 (19)	0.010 (2)	0.015 (2)
O3	0.050 (6)	0.027 (5)	0.046 (5)	0.017 (4)	0.006 (4)	0.015 (4)
C1	0.040 (6)	0.024 (5)	0.046 (6)	0.014 (5)	0.011 (5)	0.022 (5)
C2	0.028 (5)	0.023 (5)	0.029 (6)	0.017 (4)	0.009 (4)	0.022 (5)
C3	0.030 (6)	0.033 (6)	0.029 (6)	0.014 (5)	0.014 (4)	0.011 (5)
N2	0.040 (5)	0.027 (5)	0.030 (5)	0.021 (4)	0.002 (4)	0.018 (4)
C4	0.024 (5)	0.038 (6)	0.035 (6)	0.017 (5)	0.012 (4)	0.014 (5)
N1	0.039 (6)	0.029 (6)	0.034 (6)	0.021 (5)	0.004 (4)	0.013 (5)
C5	0.041 (6)	0.043 (7)	0.037 (6)	0.019 (5)	0.016 (5)	0.018 (5)

### Geometric parameters (Å, °)

**Table:**

Nd1—O2	2.526 (4)	C10—H10	0.93
Nd1—O3	2.502 (9)	C11—C12	1.485 (8)
Nd1—O5	2.392 (4)	C12—C15	1.355 (8)
Nd1—O6	2.575 (4)	C12—C13	1.431 (8)
Nd1—O9	2.341 (4)	C15—H15	0.93
Nd1—O10	2.526 (4)	C16—C17	1.391 (10)
Nd1—O1W	2.475 (4)	C16—H16	0.93
Nd1—N1^i^	2.478 (6)	C17—C18	1.366 (11)
Nd1—N7	2.638 (5)	C17—H17	0.93
Nd1—N8	2.725 (5)	C18—C19	1.361 (11)
O4—C6	1.234 (7)	C18—H18	0.93
O5—C6	1.268 (7)	C19—C20	1.419 (9)
O6—C8	1.233 (7)	C19—C21	1.431 (11)
O7—C9	1.229 (8)	C20—C24	1.427 (9)
O8—C11	1.232 (7)	C21—C22	1.330 (12)
O9—C11	1.279 (7)	C21—H21	0.93
O10—C13	1.252 (7)	C22—C23	1.424 (11)
O11—C14	1.222 (7)	C22—H22	0.93
O1W—H1W1	0.85	C23—C25	1.381 (11)
O1W—H1W2	0.85	C23—C24	1.402 (9)
N3—C10	1.351 (8)	C25—C26	1.361 (12)
N3—C9	1.366 (9)	C25—H25	0.93
N3—H3N	0.86	C26—C27	1.391 (9)
N4—C9	1.367 (8)	C26—H26	0.93
N4—C8	1.378 (7)	C27—H27	0.93
N4—H4N	0.86	O1—C1	1.238 (8)
N5—C15	1.345 (8)	O2—C1	1.191 (8)
N5—C14	1.354 (7)	O3—C3	1.307 (8)
N5—H5N	0.86	C1—C2	1.473 (8)
N6—C13	1.368 (7)	C2—C3	1.39
N6—C14	1.371 (7)	C2—C5	1.39
N6—H6N	0.86	C3—N2	1.39
N7—C16	1.305 (9)	N2—C4	1.39
N7—C20	1.375 (8)	N2—H2N	0.86
N8—C27	1.343 (9)	C4—N1	1.39
N8—C24	1.358 (8)	N1—C5	1.39
C6—C7	1.487 (8)	N1—Nd1^i^	2.478 (6)
C7—C10	1.346 (8)	C5—H5	0.93
C7—C8	1.438 (8)		
			
O2—Nd1—O3	67.5 (2)	O7—C9—N4	122.9 (7)
O2—Nd1—O5	71.6 (1)	N3—C9—N4	114.1 (6)
O2—Nd1—O6	123.5 (1)	C7—C10—N3	123.2 (6)
O2—Nd1—O9	127.7 (1)	C7—C10—H10	118.4
O2—Nd1—O10	71.8 (1)	N3—C10—H10	118.4
O2—Nd1—O1W	136.6 (2)	O8—C11—O9	124.9 (6)
O2—Nd1—N7	70.6 (2)	O8—C11—C12	118.8 (6)
O2—Nd1—N8	111.7 (2)	O9—C11—C12	116.3 (5)
O3—Nd1—O5	138.7 (2)	C15—C12—C13	117.4 (5)
O3—Nd1—O6	133.6 (3)	C15—C12—C11	119.2 (5)
O3—Nd1—O9	68.6 (3)	C13—C12—C11	123.3 (5)
O3—Nd1—O10	76.5 (3)	O10—C13—N6	116.8 (5)
O3—Nd1—O1W	138.2 (3)	O10—C13—C12	126.6 (5)
O3—Nd1—N7	87.9 (3)	N6—C13—C12	116.6 (5)
O3—Nd1—N8	65.6 (3)	O11—C14—N5	122.8 (6)
O5—Nd1—O6	67.9 (1)	O11—C14—N6	122.0 (6)
O5—Nd1—O9	139.7 (2)	N5—C14—N6	115.2 (5)
O5—Nd1—O10	85.9 (1)	N5—C15—C12	122.7 (5)
O5—Nd1—O1W	78.2 (2)	N5—C15—H15	118.7
O5—Nd1—N7	83.8 (2)	C12—C15—H15	118.7
O5—Nd1—N8	138.8 (2)	N7—C16—C17	123.6 (7)
O6—Nd1—O9	72.6 (1)	N7—C16—H16	118.2
O6—Nd1—O10	67.7 (1)	C17—C16—H16	118.2
O6—Nd1—O1W	69.0 (1)	C18—C17—C16	119.1 (8)
O6—Nd1—N7	138.2 (2)	C18—C17—H17	120.5
O6—Nd1—N8	124.8 (2)	C16—C17—H17	120.5
O9—Nd1—O10	71.5 (1)	C19—C18—C17	120.1 (7)
O9—Nd1—O1W	95.5 (2)	C19—C18—H18	120.0
O9—Nd1—N7	133.8 (2)	C17—C18—H18	120.0
O9—Nd1—N8	72.8 (2)	C18—C19—C20	118.1 (7)
O10—Nd1—O1W	136.7 (2)	C18—C19—C21	123.4 (7)
O10—Nd1—N7	142.5 (2)	C20—C19—C21	118.5 (8)
O10—Nd1—N8	135.1 (2)	N7—C20—C19	121.5 (7)
O1W—Nd1—N7	75.78 (16)	N7—C20—C24	119.0 (6)
O1W—Nd1—N8	72.93 (16)	C19—C20—C24	119.5 (6)
N7—Nd1—N8	61.14 (16)	C22—C21—C19	121.9 (7)
O9—Nd1—N1^i^	80.8 (3)	C22—C21—H21	119.1
O5—Nd1—N1^i^	124.7 (3)	C19—C21—H21	119.1
O1W—Nd1—N1^i^	147.5 (3)	C21—C22—C23	120.8 (7)
N1^i^—Nd1—O3	14.1 (3)	C21—C22—H22	119.6
N1^i^—Nd1—O10	73.0 (3)	C23—C22—H22	119.6
N1^i^—Nd1—O2	53.4 (2)	C25—C23—C24	118.0 (7)
N1^i^—Nd1—O6	137.7 (4)	C25—C23—C22	122.0 (7)
N1^i^—Nd1—N7	83.6 (4)	C24—C23—C22	119.9 (8)
N1^i^—Nd1—N8	75.1 (3)	N8—C24—C23	122.7 (7)
C6—O5—Nd1	140.6 (4)	N8—C24—C20	117.9 (5)
C8—O6—Nd1	123.2 (4)	C23—C24—C20	119.4 (6)
C11—O9—Nd1	137.2 (4)	C26—C25—C23	119.8 (7)
C13—O10—Nd1	130.2 (4)	C26—C25—H25	120.1
Nd1—O1W—H1W1	117.9	C23—C25—H25	120.1
Nd1—O1W—H1W2	107.8	C25—C26—C27	119.5 (8)
H1W1—O1W—H1W2	107.7	C25—C26—H26	120.2
C10—N3—C9	122.5 (6)	C27—C26—H26	120.2
C10—N3—H3N	118.7	N8—C27—C26	122.6 (7)
C9—N3—H3N	118.7	N8—C27—H27	118.7
C9—N4—C8	126.9 (6)	C26—C27—H27	118.7
C9—N4—H4N	116.5	C1—O2—Nd1	143.9 (5)
C8—N4—H4N	116.5	C3—O3—Nd1	135.9 (6)
C15—N5—C14	122.6 (5)	O2—C1—O1	120.4 (8)
C15—N5—H5N	118.7	O2—C1—C2	119.1 (7)
C14—N5—H5N	118.7	O1—C1—C2	120.5 (8)
C13—N6—C14	125.4 (5)	C3—C2—C5	120.0
C13—N6—H6N	117.3	C3—C2—C1	124.7 (6)
C14—N6—H6N	117.3	C5—C2—C1	115.3 (6)
C16—N7—C20	117.7 (6)	O3—C3—N2	113.1 (6)
C16—N7—Nd1	120.3 (4)	O3—C3—C2	126.9 (6)
C20—N7—Nd1	121.9 (4)	N2—C3—C2	120.0
C27—N8—C24	117.3 (6)	C4—N2—C3	120.0
C27—N8—Nd1	122.7 (4)	C4—N2—H2N	120.0
C24—N8—Nd1	119.9 (4)	C3—N2—H2N	120.0
O4—C6—O5	123.3 (6)	N2—C4—N1	120.0
O4—C6—C7	118.8 (5)	N2—C4—Nd1^i^	175.4 (3)
O5—C6—C7	117.9 (5)	N1—C4—Nd1^i^	55.6 (3)
C10—C7—C8	117.7 (6)	C5—N1—C4	120.0
C10—C7—C6	119.6 (5)	C5—N1—Nd1^i^	143.1 (4)
C8—C7—C6	122.7 (5)	C4—N1—Nd1^i^	96.8 (4)
O6—C8—N4	118.1 (5)	N1—C5—C2	120.0
O6—C8—C7	126.6 (5)	N1—C5—H5	120.0
N4—C8—C7	115.3 (5)	C2—C5—H5	120.0
O7—C9—N3	123.0 (7)		
			
O9—Nd1—O5—C6	21.5 (8)	C9—N3—C10—C7	−0.6 (11)
O1W—Nd1—O5—C6	106.0 (7)	Nd1—O9—C11—O8	−131.1 (6)
N1^i^—Nd1—O5—C6	−99.5 (8)	Nd1—O9—C11—C12	50.7 (8)
O3—Nd1—O5—C6	−97.6 (8)	O8—C11—C12—C15	−30.9 (9)
O10—Nd1—O5—C6	−33.4 (6)	O9—C11—C12—C15	147.5 (6)
O2—Nd1—O5—C6	−105.6 (7)	O8—C11—C12—C13	153.3 (6)
O6—Nd1—O5—C6	34.1 (6)	O9—C11—C12—C13	−28.4 (9)
N7—Nd1—O5—C6	−177.3 (7)	Nd1—O10—C13—N6	−156.8 (4)
N8—Nd1—O5—C6	152.1 (6)	Nd1—O10—C13—C12	24.7 (9)
C4^i^—Nd1—O5—C6	−104.4 (7)	C14—N6—C13—O10	179.7 (6)
O9—Nd1—O6—C8	125.4 (5)	C14—N6—C13—C12	−1.7 (9)
O5—Nd1—O6—C8	−46.1 (4)	C15—C12—C13—O10	177.3 (6)
O1W—Nd1—O6—C8	−131.5 (5)	C11—C12—C13—O10	−6.8 (10)
N1^i^—Nd1—O6—C8	71.6 (6)	C15—C12—C13—N6	−1.2 (8)
O3—Nd1—O6—C8	90.9 (5)	C11—C12—C13—N6	174.7 (5)
O10—Nd1—O6—C8	48.7 (4)	C15—N5—C14—O11	178.8 (7)
O2—Nd1—O6—C8	1.2 (5)	C15—N5—C14—N6	−1.8 (10)
N7—Nd1—O6—C8	−97.0 (5)	C13—N6—C14—O11	−177.5 (6)
N8—Nd1—O6—C8	179.0 (4)	C13—N6—C14—N5	3.1 (10)
C4^i^—Nd1—O6—C8	30.3 (6)	C14—N5—C15—C12	−1.0 (10)
O5—Nd1—O9—C11	−89.4 (6)	C13—C12—C15—N5	2.5 (9)
O1W—Nd1—O9—C11	−167.6 (6)	C11—C12—C15—N5	−173.6 (6)
N1^i^—Nd1—O9—C11	45.0 (6)	C20—N7—C16—C17	−0.5 (10)
O3—Nd1—O9—C11	52.3 (6)	Nd1—N7—C16—C17	−179.3 (6)
O10—Nd1—O9—C11	−30.0 (6)	N7—C16—C17—C18	0.2 (12)
O2—Nd1—O9—C11	17.6 (6)	C16—C17—C18—C19	0.2 (12)
O6—Nd1—O9—C11	−101.6 (6)	C17—C18—C19—C20	−0.2 (11)
N7—Nd1—O9—C11	116.9 (6)	C17—C18—C19—C21	178.3 (8)
N8—Nd1—O9—C11	122.2 (6)	C16—N7—C20—C19	0.5 (9)
C4^i^—Nd1—O9—C11	33.9 (6)	Nd1—N7—C20—C19	179.3 (5)
O9—Nd1—O10—C13	−10.0 (5)	C16—N7—C20—C24	179.6 (6)
O5—Nd1—O10—C13	136.0 (5)	Nd1—N7—C20—C24	−1.6 (8)
O1W—Nd1—O10—C13	68.0 (6)	C18—C19—C20—N7	−0.1 (10)
N1^i^—Nd1—O10—C13	−95.8 (6)	C21—C19—C20—N7	−178.7 (6)
O3—Nd1—O10—C13	−81.7 (5)	C18—C19—C20—C24	−179.3 (7)
O2—Nd1—O10—C13	−152.0 (5)	C21—C19—C20—C24	2.2 (10)
O6—Nd1—O10—C13	68.3 (5)	C18—C19—C21—C22	−179.8 (8)
N7—Nd1—O10—C13	−149.7 (5)	C20—C19—C21—C22	−1.3 (12)
N8—Nd1—O10—C13	−49.2 (6)	C19—C21—C22—C23	−1.2 (13)
C4^i^—Nd1—O10—C13	−124.7 (5)	C21—C22—C23—C25	−176.1 (8)
O9—Nd1—N7—C16	−173.0 (5)	C21—C22—C23—C24	2.8 (12)
O5—Nd1—N7—C16	23.8 (5)	C27—N8—C24—C23	1.0 (10)
O1W—Nd1—N7—C16	103.2 (5)	Nd1—N8—C24—C23	−177.9 (5)
N1^i^—Nd1—N7—C16	−102.2 (6)	C27—N8—C24—C20	−176.9 (6)
O3—Nd1—N7—C16	−115.7 (5)	Nd1—N8—C24—C20	4.2 (8)
O10—Nd1—N7—C16	−51.2 (6)	C25—C23—C24—N8	−0.8 (10)
O2—Nd1—N7—C16	−48.8 (5)	C22—C23—C24—N8	−179.7 (7)
O6—Nd1—N7—C16	70.1 (6)	C25—C23—C24—C20	177.1 (7)
N8—Nd1—N7—C16	−178.7 (6)	C22—C23—C24—C20	−1.8 (10)
C4^i^—Nd1—N7—C16	−75.8 (5)	N7—C20—C24—N8	−1.8 (9)
O9—Nd1—N7—C20	8.2 (6)	C19—C20—C24—N8	177.3 (6)
O5—Nd1—N7—C20	−155.0 (5)	N7—C20—C24—C23	−179.8 (6)
O1W—Nd1—N7—C20	−75.6 (5)	C19—C20—C24—C23	−0.6 (9)
N1^i^—Nd1—N7—C20	79.0 (5)	C24—C23—C25—C26	−0.7 (11)
O3—Nd1—N7—C20	65.6 (5)	C22—C23—C25—C26	178.2 (8)
O10—Nd1—N7—C20	130.0 (4)	C23—C25—C26—C27	1.9 (12)
O2—Nd1—N7—C20	132.4 (5)	C24—N8—C27—C26	0.3 (11)
O6—Nd1—N7—C20	−108.7 (5)	Nd1—N8—C27—C26	179.2 (6)
N8—Nd1—N7—C20	2.5 (4)	C25—C26—C27—N8	−1.8 (12)
C4^i^—Nd1—N7—C20	105.4 (5)	O9—Nd1—O2—C1	50.4 (6)
O9—Nd1—N8—C27	2.1 (5)	O5—Nd1—O2—C1	−170.3 (6)
O5—Nd1—N8—C27	−147.0 (5)	O1W—Nd1—O2—C1	−122.0 (6)
O1W—Nd1—N8—C27	−99.4 (6)	N1^i^—Nd1—O2—C1	16.0 (8)
N1^i^—Nd1—N8—C27	86.9 (6)	O3—Nd1—O2—C1	15.5 (6)
O3—Nd1—N8—C27	75.9 (6)	O10—Nd1—O2—C1	98.0 (6)
O10—Nd1—N8—C27	40.9 (6)	O6—Nd1—O2—C1	143.8 (6)
O2—Nd1—N8—C27	126.6 (5)	N7—Nd1—O2—C1	−80.5 (6)
O6—Nd1—N8—C27	−51.4 (6)	N8—Nd1—O2—C1	−34.2 (6)
N7—Nd1—N8—C27	177.7 (6)	C4^i^—Nd1—O2—C1	12.6 (9)
C4^i^—Nd1—N8—C27	108.0 (6)	O9—Nd1—O3—C3	−151.4 (14)
O9—Nd1—N8—C24	−179.1 (5)	O5—Nd1—O3—C3	−8.8 (16)
O5—Nd1—N8—C24	31.9 (6)	O1W—Nd1—O3—C3	135.1 (11)
O1W—Nd1—N8—C24	79.4 (5)	N1^i^—Nd1—O3—C3	−2(2)
N1^i^—Nd1—N8—C24	−94.3 (6)	O10—Nd1—O3—C3	−76.2 (13)
O3—Nd1—N8—C24	−105.3 (5)	O2—Nd1—O3—C3	−0.5 (12)
O10—Nd1—N8—C24	−140.3 (4)	O6—Nd1—O3—C3	−116.0 (12)
O2—Nd1—N8—C24	−54.6 (5)	N7—Nd1—O3—C3	69.4 (13)
O6—Nd1—N8—C24	127.4 (4)	N8—Nd1—O3—C3	128.4 (14)
N7—Nd1—N8—C24	−3.4 (4)	C4^i^—Nd1—O3—C3	1.4 (13)
C4^i^—Nd1—N8—C24	−73.2 (5)	Nd1—O2—C1—O1	158.2 (7)
Nd1—O5—C6—O4	168.0 (5)	Nd1—O2—C1—C2	−21.9 (7)
Nd1—O5—C6—C7	−12.1 (9)	O2—C1—C2—C3	10.7 (6)
O4—C6—C7—C10	−19.2 (9)	O1—C1—C2—C3	−169.4 (6)
O5—C6—C7—C10	160.9 (6)	O2—C1—C2—C5	−168.7 (5)
O4—C6—C7—C8	160.7 (6)	O1—C1—C2—C5	11.2 (5)
O5—C6—C7—C8	−19.2 (8)	Nd1—O3—C3—N2	175.8 (8)
Nd1—O6—C8—N4	−137.4 (5)	Nd1—O3—C3—C2	−3.7 (18)
Nd1—O6—C8—C7	44.3 (8)	C5—C2—C3—O3	179.5 (12)
C9—N4—C8—O6	177.5 (7)	C1—C2—C3—O3	0.1 (11)
C9—N4—C8—C7	−4.0 (10)	C5—C2—C3—N2	0.0
C10—C7—C8—O6	178.5 (6)	C1—C2—C3—N2	−179.4 (6)
C6—C7—C8—O6	−1.3 (9)	O3—C3—N2—C4	−179.6 (11)
C10—C7—C8—N4	0.2 (8)	C2—C3—N2—C4	0.0
C6—C7—C8—N4	−179.7 (5)	C3—N2—C4—N1	0.0
C10—N3—C9—O7	175.9 (8)	N2—C4—N1—C5	0.0
C10—N3—C9—N4	−2.7 (11)	N2—C4—N1—Nd1^i^	−178.2 (5)
C8—N4—C9—O7	−173.4 (8)	C4—N1—C5—C2	0.0
C8—N4—C9—N3	5.2 (11)	Nd1^i^—N1—C5—C2	177.1 (8)
C8—C7—C10—N3	1.9 (10)	C3—C2—C5—N1	0.0
C6—C7—C10—N3	−178.2 (6)	C1—C2—C5—N1	179.4 (6)

Symmetry codes: (i) −*x*+1, −*y*+1, −*z*+1.

### Hydrogen-bond geometry (Å, °)

**Table:**

*D*—H···*A*	*D*—H	H···*A*	*D*···*A*	*D*—H···*A*
N3—H3N···O1^ii^	0.86	2.19	2.877 (13)	136
N3—H3N···O2^ii^	0.86	2.10	2.883 (7)	152
N4—H4N···O8^iii^	0.86	1.91	2.768 (7)	175
N5—H5N···O11^iv^	0.86	1.95	2.786 (7)	164
N6—H6N···O4^ii^	0.86	1.86	2.709 (6)	167
O1W—H1W1···O4^v^	0.85	2.04	2.822 (7)	153
O1W—H1W2···O11^vi^	0.85	2.14	2.921 (6)	153

Symmetry codes: (ii) −*x*+1, −*y*, −*z*; (iii) −*x*+1, −*y*+1, −*z*; (iv) −*x*+2, −*y*+1, −*z*; (v) −*x*, −*y*, −*z*; (vi) *x*−1, *y*, *z*.
